# *FKS1* Is Required for Cryptococcus neoformans Fitness *In Vivo*: Application of Copper-Regulated Gene Expression to Mouse Models of Cryptococcosis

**DOI:** 10.1128/msphere.00163-22

**Published:** 2022-05-04

**Authors:** Sarah R. Beattie, Andrew J. Jezewski, Laura C. Ristow, Melanie Wellington, Damian J. Krysan

**Affiliations:** a Department of Pediatrics, Carver College of Medicine, University of Iowa, Iowa City, Iowa, USA; b Department of Microbiology and Immunology, Carver College of Medicine, University of Iowa, Iowa City, Iowa, USA; Yonsei University

**Keywords:** *Cryptococcus neoformans*, echinocandins, essential genes, glucan synthase

## Abstract

There is an urgent need for new antifungals to treat cryptococcal meningoencephalitis, a leading cause of mortality in people living with HIV/AIDS. An important aspect of antifungal drug development is the validation of targets to determine whether they are required for the survival of the organism in animal models of disease. In Cryptococcus neoformans, a copper-regulated promoter (pCTR4-2) has been used previously to modulate gene expression *in vivo*. The premise for these experiments is that copper concentrations differ depending on the host niche. Here, we directly test this premise and confirm that the expression of *CTR4*, the promoter used to regulate gene expression, is much lower in the mouse lung compared to the brain. To further explore this approach, we applied it to the gene encoding 1,3-β-glucan synthase, *FKS1*. *In vitro*, reduced expression of *FKS1* has little effect on growth but does activate the cell wall integrity stress response and increase susceptibility to caspofungin, a direct inhibitor of Fks1. These data suggest that compensatory pathways that reduce C. neoformans resistance do so through posttranscriptional effects. *In vivo*, however, a less pronounced reduction in *FKS1* expression leads to a much more significant reduction in lung fungal burden (~1 log_10_ CFU), indicating that the compensatory responses to a reduction in *FKS1* expression are not as effective *in vivo* as they are *in vitro*. In summary, use of copper-regulated expression of putative drug targets *in vitro* and *in vivo* can provide insights into the biological consequences of reduced activity of the target during infection.

**IMPORTANCE** Conditional expression systems are widely used to genetically validate antifungal drug targets in mouse models of infection. Copper-regulated expression using the promoter of the *CTR4* gene has been sporadically used for this purpose in C. neoformans. Here, we show that *CTR4* expression is low in the lung and high in the brain, establishing the basic premise behind this approach. We applied the approach to the study of *FKS1*, the gene encoding the target of the echinocandin class of 1,3-β-glucan synthase inhibitors. Our *in vitro* and *in vivo* studies indicate that C. neoformans tolerates extremely low levels of *FKS1* expression. This observation provides a potential explanation for the poor activity of 1,3-β-glucan synthase inhibitors toward C. neoformans.

## INTRODUCTION

Cryptococcus species are among the most important human fungal pathogens and have global effects on human health, particularly for people living with HIV/AIDS (1, 2). Serological surveillance suggest that most people have been exposed to Cryptococcus early in life (3). For the vast majority, this exposure does not lead to disease. However, individuals with altered T-cell function, and indeed, some with apparently normal immune function, develop cryptococcal meningoencephalitis (CME). CME is uniformly fatal unless treated and is a leading cause of death for people living with HIV/AIDS (4). Importantly, CME can be the sentinel event leading to the diagnosis of HIV infection. As a result, many people must first survive CME to take advantage of the lifesaving advances in the treatment of HIV.

The first-line regimens for the treatment of cryptococcal meningitis are currently based on various combinations of amphotericin B, flucytosine, and/or fluconazole (5). Unfortunately, these medications all have issues that limit their effectiveness and/or utility. Amphotericin B is toxic, requires intravenous administration, and necessitates laboratory monitoring of electrolytes (6); these characteristics can make amphotericin logistically difficult to use in resource-limited regions without extensive medical infrastructure. Flucytosine is also toxic and is not available in many of the countries that have the highest rates of disease (7). Finally, fluconazole is inexpensive and available, but its efficacy is poor as a single agent and it must be combined with either amphotericin B or flucytosine in order to have a reasonable outcome. The development of safe and effective therapies for CME that are accessible to people who live in the areas with the highest rates of disease should be a high priority for modern medicine.

To address the unmet clinical need for new anticryptococcal therapies, a number of groups have explored repurposing and new chemical entity discovery approaches based on both high-throughput phenotypic screening and target-focused medicinal chemistry campaigns (8). A key task during preclinical drug development is to validate that a putative target leads to reduced growth or virulence of the fungus in an animal model of infection. Usually, these targets are encoded by genes that are essential for viability making genetic studies more complicated, particularly in assessing the contribution to virulence. The most common approach to evaluate essential genes in models of fungal infection is the construction of conditional expression alleles that are responsive to tetracycline/doxycycline, the so-called Tet_OFF_ technology (9–11). A major advantage to this system is the ability to administer doxycycline to mice, which can then regulate fungal gene expression *in vivo* (12–14). To date, this technology has not been applicable to C. neoformans (15). Modulation of some genes has been achieved, but it requires extremely high doses of doxycycline that are not achievable in mice. The reasons for this technical limitation are not clear, but similar problems have been reported for the application of doxycycline regulation to Ustilago maydis, another basidiomycete (16). Thus, in C. neoformans, genetic validation studies have been largely limited to either *in vitro* studies using alternative conditional promoters or *in vivo* analysis of nonessential genes.

One of the most widely used system for the conditional expression of genes in C. neoformans is based on the promoter for the copper transporter, *CTR4*. Initially developed by the Doering lab, replacement of the promoter for the gene of interest with multiple copies of the *CTR4* (p*CTR4-2*) leads to an allele that is highly expressed at low copper concentrations and repressed in the presence of high concentrations of copper (17). Prior to our recent work (18), there had been one report of using a *CTR4*-regulated gene to assess gene function in the setting of a mouse model of cryptococcal infection. In this work, the promoters of the essential fatty acid synthases *FAS1*/*FAS2* were replaced by p*CTR4-2* (15). *In vitro*, neither strain was able to grow in the presence of 25 μM copper sulfate, while only the p*CTR4-2-FAS2* strain showed reduced fungal burden in the lungs compared to that of the H99 strain following intranasal infection. No dissemination to the brain was detected with the p*CTR4-2-FAS2* strain, while the p*CTR4-2-FAS1* strain showed an ~1 log_10_ reduction in brain burden. These observations are consistent with the likelihood that Cryptococcus cells in the lung experience a relatively high copper concentration, leading to reduced expression of *FAS2* and reduced fungal burden. In addition, the discordance between the *in vitro* and *in vivo* phenotypes of the p*CTR4*-2-*FAS1* strain suggest that the effect of p*CTR4-2* regulation of gene expression may differ with the specific gene under regulation.

Recently, we reported that infection of mice with a strain that contained *HSP90* under the control of p*CTR4-2* lead to reduced expression of *HSP90* in the lung relative to that in the congenic control, whereas the same strain inoculated intravenously and sampled from the brain had dramatically increased expression relative to that in the control (18). Consistent with these expression data, mice infected intranasally with the p*CTR4-2-HSP90* strain survived longer than those infected with the wild type, and there was no difference in survival between the two strains in the intravenous inoculation experiment. These data indicate that C. neoformans experiences high copper conditions in the lung and low copper conditions in the brain and that p*CTR4-2*-regulated alleles might be useful to validate drug targets or study essential gene function in mouse models of cryptococcosis. Here, we directly measure the expression of C. neoformans *CTR4* in infected lung and brain tissue and confirm that it is much lower in the lung than in the brain, supporting the previous observations and the utility of this approach for some genes.

To further explore the use of p*CTR4-2* in the regulation of essential genes related to antifungal therapy, we generated a *pCTR4-2-FKS1* strain and examined its *in vitro* and *in vivo* phenotypes. Fks1 is a 1,3-β-glucan synthase and the target of the echinocandin class of antifungal drugs (19). Echinocandins such as caspofungin have poor activity against C. neoformans and are ineffective in mouse models of infection (20). This ineffectiveness is despite genetic evidence indicating that *FKS1* is an essential gene (21) and biochemical experiments indicating that Fks1 is inhibited by echinocandins (22). The mechanism of echinocandin ineffectiveness against C. neoformans is unclear and remains an area of active investigation. Here, we show that *in vitro*, C. neoformans tolerates drastic reductions in *FKS1* expression with minimal changes in growth, namely, a modest increase in echinocandin susceptibility and activation of the cell wall integrity response. *In vivo*, the fitness of a *pCTR4-2-FKS1* strain is reduced in the lung to a greater extent than that observed *in vitro*. These data suggest that C. neoformans is remarkably resistant to reduction of Fks1 activity *in vitro* but is somewhat more susceptible *in vivo*.

## RESULTS

### C. neoformans cells infecting the brain have elevated *CTR4* expression relative to that in cells infecting the lung.

Pioneering studies of copper homeostasis in C. neoformans during infection by Waterman et al. indicated that in C. neoformans copper is limiting during infection of macrophages and the central nervous system (CNS), but not during pulmonary infection (23). Ding et al. used bioluminescence to show that expression of the metallothionein *CMT1*, which is induced under high copper concentrations, is relatively elevated in the lung, while *CTR4* is detectable but expressed at a relatively low level (24). The elevated ratio of *CMT1* to *CTR4* is consistent with C. neoformans occupying a niche in the lung that is relatively copper replete. Further supporting that conclusion, Waterman et al. also found that deletion of *CUF1*, the gene encoding a transcription factor that activates *CTR4* expression, had no effect on replication of C. neoformans in the lung but prevented dissemination to the brain (23). Although *CTR4* expression appears to differ between C. neoformans cells infecting the lung and the brain of mice, it is required for virulence in both pulmonary and intravenous inoculation models. The differential expression data suggested that it might be possible to use *CTR4-2*-regulated alleles as knockdown alleles to study essential or severely compromised mutants during pulmonary infection; alternatively, during CNS infection, the construct could display features of overexpression.

**FIG 1:**
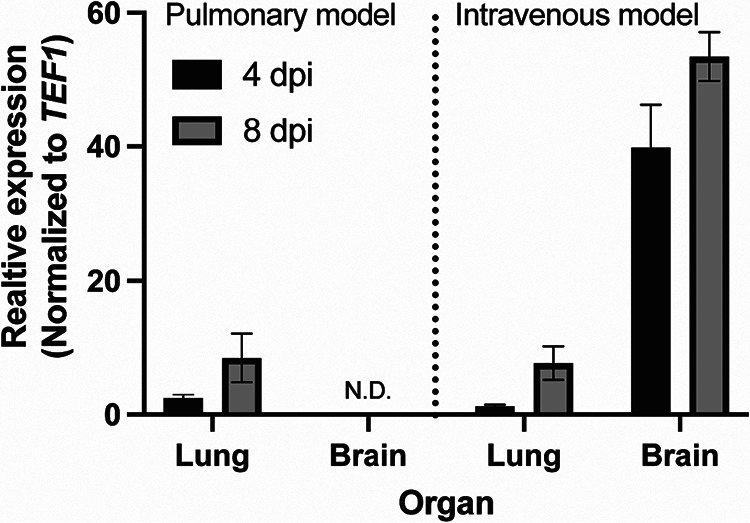
Expression of the copper-regulated transporter *CTR4* is higher in the brain than in the lungs. Brains and lungs were harvested from mice at 4 or 8 days postinoculation (dpi) with KN99α via intranasal instillation (pulmonary model) or by lateral tail vein injection (intravenous model). Data represent mean and standard error of the mean (SEM) of three mice per group. ND, none detected.

To our knowledge, the expression of *CTR4* in C. neoformans during infection of mouse lung or brain has not been directly characterized using reverse transcription-PCR (RT-PCR); previous data were derived from experiments using either fluorescent or luminescent *CTR4* fusion reporters. To directly measure *CTR4* message *in vivo*, we inoculated AJ/cr mice with the reference strain KN99α using the intranasal and the intravenous inoculation routes; to reiterate, the intravenous route establishes CNS and pulmonary infection within hours, while the intranasal route immediately establishes pulmonary infection but infection can take over a week to disseminate to the brain. We harvested lungs and brains from animals inoculated by each route on days 4 and 8 postinoculation. The expression of *CTR4* in the lungs was the same between the intravenous and intranasal models; on day 4, expression was low, and it increased slightly by day 8 in both models (Fig. 1). We did not detect any *CTR4* or *TEF1* expression in brain samples from the intranasal model at either 4 or 8 days postinoculation (dpi), suggesting that these time points occur before significant dissemination to the brain. In the brains collected from intravenously inoculated animals, we observed much higher expression of *CTR4*. Compared to that in the lungs, the expression of *CTR4* in the brain was 40-fold and 7-fold higher at days 4 and 8, respectively (Fig. 1). These data clearly show that the brain is copper replete relative to the lung, consistent with results of previous reporter-based assays. These data are also consistent with the relative expression of *HSP90* from the p*CTR4-2-HSP90* strain (18) in the lung and brain and support the concept that pC*TR4-2* may be applicable to modulating the expression of C. neoformans genes during mouse infection.

### Application of p*CTR4-2* to the 1,3-β-glucan synthase catalytic subunit, *FKS1*.

The echinocandin class of antifungal drugs is the first to directly target the fungal cell wall and is the therapy of choice for invasive Candida infections (25). Echinocandins are also active against Aspergillus fumigatus and cause lysis of hyphal compartments, leading to fungistatic activity (26). The MIC of echinocandins against C. neoformans is much higher than the MIC for *Candida* spp. or the minimum effective concentration (MEC) for Aspergillus spp. A typical MIC for caspofungin against C. neoformans is 16 μg/mL, while MICs for susceptible *Candida* and Aspergillus species are 10- to 1,000-fold lower (27). The mechanism for the reduced susceptibility of C. neoformans to echinocandins is the subject of active investigation (28–31). Although recent work has shed light onto genes and processes that contribute to this resistance, its fundamental molecular basis remains to be described. The C. neoformans Fks1 is inhibited by echinocandins *in vitro* (22), and the lack of successful attempts to generate deletion mutants indicates that *FKS1* is essential (21). To further confirm the latter finding and to test the effect of reduced *FKS1* expression on the biology of C. neoformans and its fitness during mouse infection, we constructed a strain containing the p*CTR4-2-FKS1* allele.

**FIG 2:**
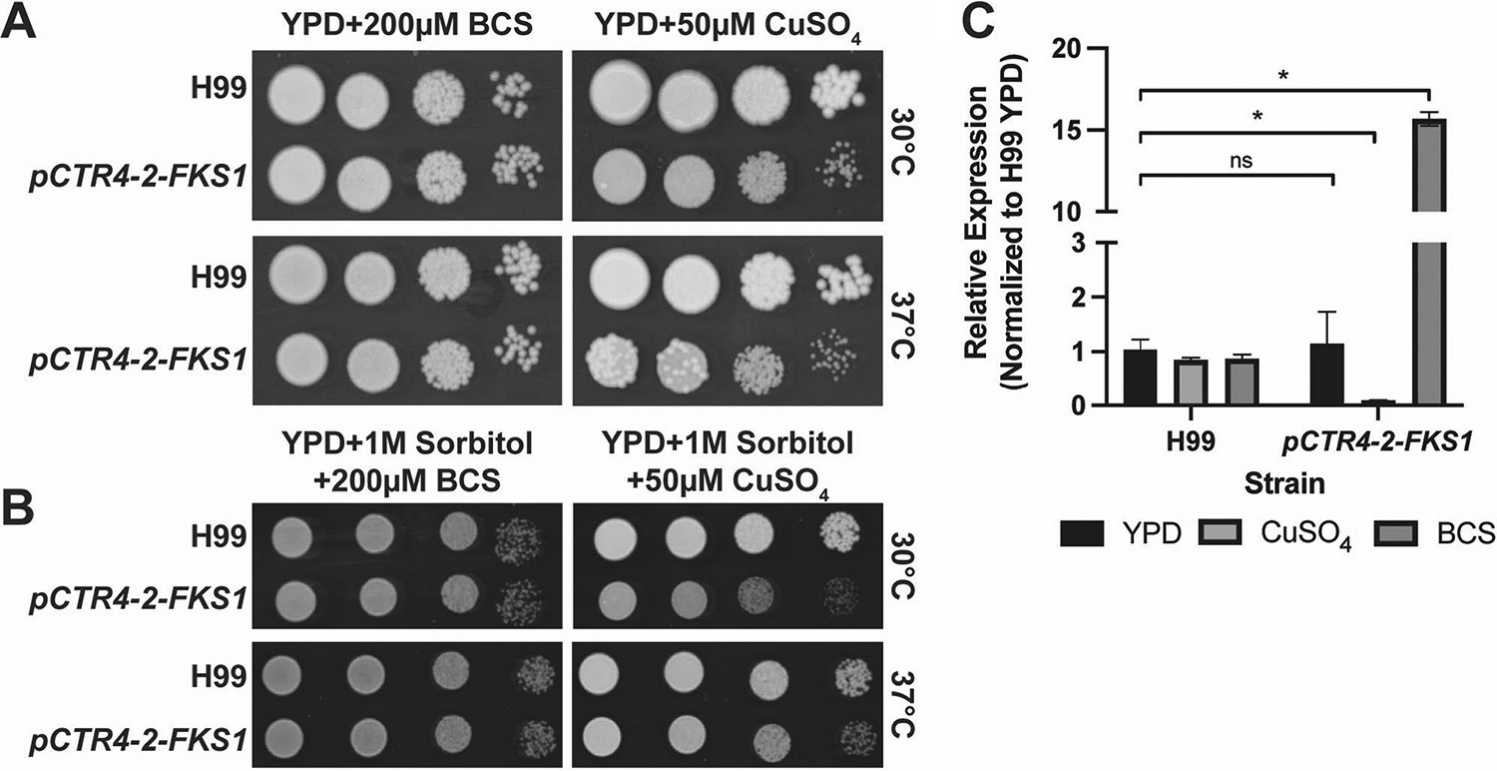
Expression of *FKS1* in the *pCTR4-2-FKS1* strain responds to copper. Tenfold serial dilutions of the H99 and *pCTR4-2-FKS1* strains on yeast extract-peptone-dextrose (YPD) (A) or YPD plus 1 M sorbitol (B) supplemented with 200 μM bovine calf serum (BCS) or 50 μM CuSO_4_. Plates were incubated at 30°C or 37°C for 72 h. (C) Expression of *FKS1* in the *pCTR4-2-FKS1* strain is reduced 10-fold in YPD and with 50 μM copper sulfate and is overexpressed with the addition of 200 μM BCS. Data represent mean and SEM of 3 biological replicates. *, *P* < 0.007; n.s., not significant by unpaired *t test*, compared to H99 YPD.

To do so, we utilized the recently optimized CRISPR/Cas9 system reported by Huang et al. to knock-in in the p*CTR4-2* construct immediately upstream of the start codon (32). On yeast extract-peptone-dextrose (YPD) and medium containing bathocuproine sulphonate (BCS), two independent strains grew similarly to the H99 parental strain at 37°C (Fig. 2A; data not shown). In high-copper medium (50 μM CuSO_4_), the p*CTR4-2-FKS1* strain showed a modest reduction in growth relative to that of the parental strain. The growth defect is less apparent when the strains are incubated at 30°C (Fig. 2A) and is not exacerbated with higher copper concentrations (up to 200 μM; data not shown). Elevated temperature causes cell wall stress in C. neoformans and activates the cell wall integrity MAP kinase pathway (33). Notably, we observed the appearance of isolated suppressor colonies when the p*CTR4-2-FKS1* strain was grown at 37°C on YPD supplemented with copper, the p*CTR4-2-FKS1* strain (Fig. 2A). These larger colonies did not emerge when H99 was incubated under the same conditions. Finally, we tested whether the addition of sorbitol, an osmotic stabilizer, could rescue the growth defect of the p*CTR4-2-FKS1* strain in the presence of copper. Although we did not observe any rescue of the overall growth of the p*CTR4-2-FKS1* strain on YPD supplemented with 1 M sorbitol and 50 μM CuSO_4_, the generation of suppressors was eliminated (Fig. 2B), suggesting that under these conditions, this strain experiences significant cell wall stress, which is reduced when grown on an osmotic stabilizer.

The relative insensitivity of the p*CTR4-2-FKS1* strain to copper addition could be due to intrinsically low expression of *FKS1*, to incomplete suppression, or to very little *FKS1* expression being required for replication. To determine if any of these explanations were operative, we compared *FKS1* expression in YPD, YPD plus BCS, and YPD plus 50 μM CuSO_4_, the same conditions under which the growth assays were performed. As shown in Fig. 2C, the expression of *FKS1* is reduced by 100-fold in copper-supplemented medium relative to that in the parental H99 strain. Conversely, the addition of BCS restored *FKS1* expression to levels 15-fold higher than those in H99, suggesting that in YPD, the overexpression of *FKS1* does not confer any growth defects at 30°C or 37°C. Compared to those of our growth assays, these results are striking. Whereas the suppression of other essential genes, such as *FAS1* or *FAS2*, results in virtually no growth at 25 μM copper (15), the suppression of *FKS1* by 100-fold causes only a modest growth defect (Fig. 2A and B). These data suggest that C. neoformans can withstand a significant reduction in *FKS1* expression without severe effects on fitness.

### Reduced expression of *FKS1* results in activation of the cell wall integrity pathway and remodeling of the cell wall.

**FIG 3:**
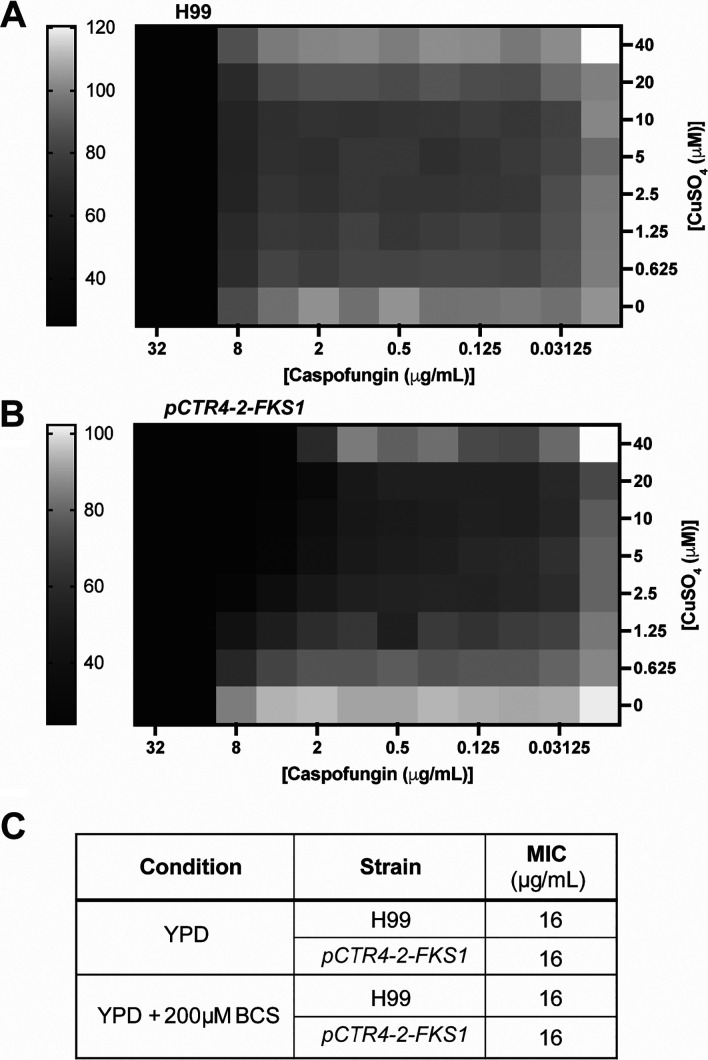
Copper increases the sensitivity of the *pCTR4-2-FKS1* strain to caspofungin. Fractional inhibitory concentration (FIC) checkerboard assay with the H99 (A) or *pCTR4-2-FKS1* (B) strains with increasing concentrations of caspofungin and copper sulfate. Heatmaps represent percent growth compared to growth of the untreated control as determined by optical density at 600 nm (OD_600_) reading after 48 h at 37°C. Representative plates of two independent replicates. (C) Caspofungin MICs in YPD or YPD supplemented with 200 μM BCS.

Although copper-containing medium modestly reduces the growth of the p*CTR4-2-FKS1* strain, the overall viability is striking considering that expression of this essential gene is reduced by 100-fold. To determine whether this significant reduction in *FKS1* expression results in a functional reduction of activity, we examined the effect of copper repression on the susceptibility of the p*CTR4-2-FKS1* strain to caspofungin. We performed checkerboard assays with increasing concentrations of caspofungin and copper sulfate (Fig. 3A and B). In the absence of copper, the MIC for the p*CTR4-2-FKS1* strain is identical to that for H99 (Fig. 3C). The MIC for H99 is 16 μg/mL, and this is unaffected by increased concentrations of copper (Fig. 3A). However, the MIC for the p*CTR4-2-FKS1* strains decreases 4-fold to 4 μg/mL in the presence of 40 μM copper sulfate (Fig. 3B). Overexpression of *FKS1* with the addition of BCS did not change the caspofungin MIC compared to that for H99 (Fig. 3C), suggesting that increased target abundance is not enough to drive increased caspofungin resistance.

**FIG 4:**
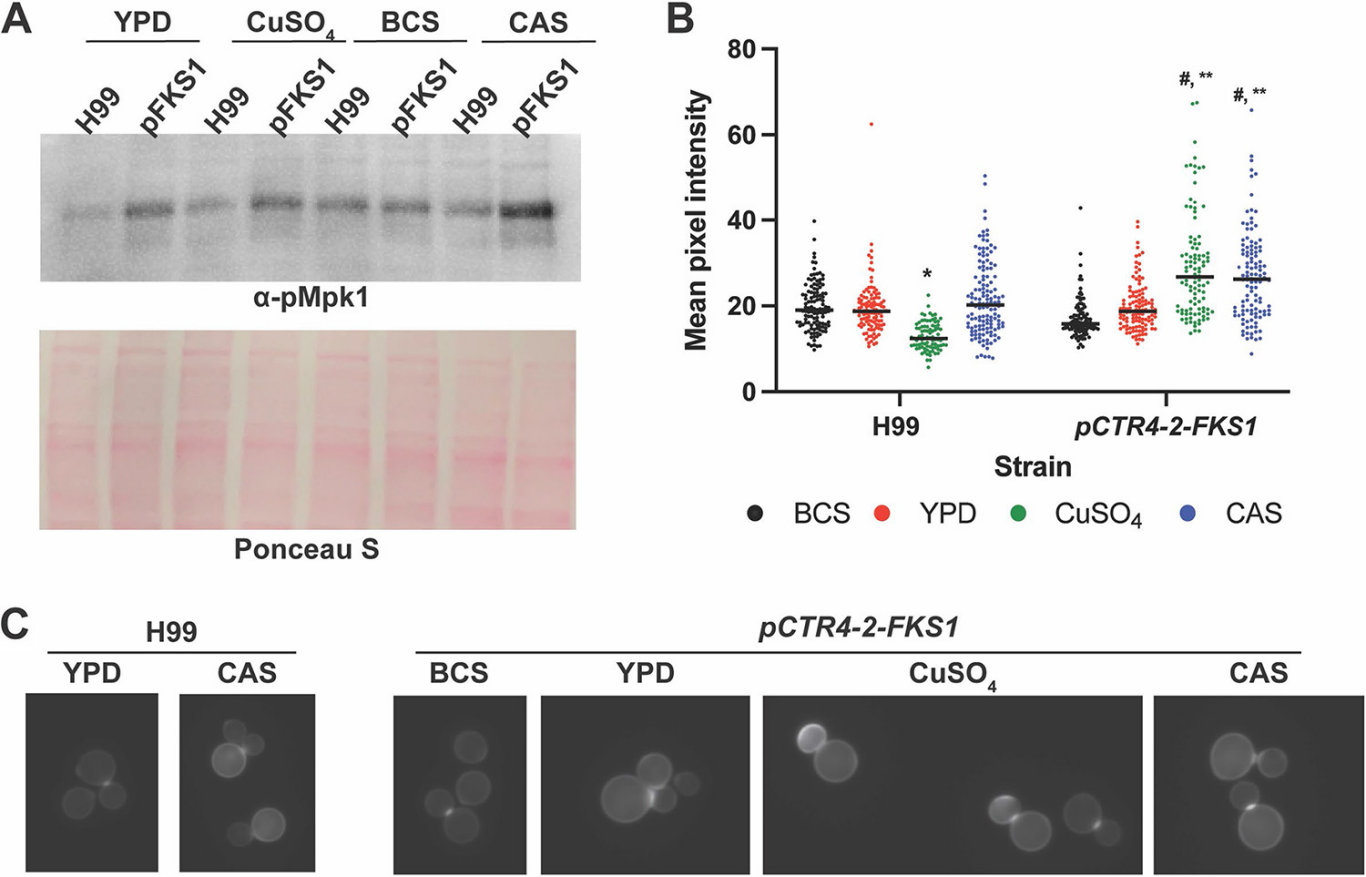
Knockdown of *FKS1* results in activation of the cell wall integrity pathway (CWIP) and an increase in chitin in the cell wall. (A) Western blotting of phosphorylated Mpk1 (pMpk1) (indicated with arrow) after the indicated treatments. Ponceau S shown as a loading control. (B) Mean pixel intensity of calcofluor white (CFW)-stained cells, quantified using ImageJ. Representative data from two independent experiments with at least 100 cells per condition. Line represents median value. *, **, #, *P* < 0.0001 compared to the H99 strain in YPD (*) or the *pCTR4-2-FKS1* strain in YPD (**) or BCS (#) by one-way analysis of variance (ANOVA) with Tukey’s multiple-comparisons test. (C) Representative images of CFW-stained cells. BCS, 200 μM BCS; CuSO_4_, 50 μM copper sulfate; CAS, 8 μg/mL caspofungin.

Reduction in Fks1 activity with caspofungin treatment activates of the cell wall integrity pathway (CWIP), resulting in a compensatory increase in chitin (30, 34). Thus, we hypothesized that our p*CTR4-2-FKS1* strain would have a similar response when grown under repressive conditions. To test this, we incubated H99 or the p*CTR4-2-FKS1* strain in YPD or in YPD supplemented with 50 μM copper sulfate, 200 μM BCS, or a subinhibitory concentration of caspofungin (8 μg/mL) to the mid-log phase and then performed Western blot analysis for phosphorylated Mpk1 (pMpk1). Mpk1 is the terminal MAP kinase in the CWIP and is phosphorylated in response to cell wall stresses such as exposure to caspofungin (35). When incubated in either YPD or YPD supplemented with copper sulfate, Mpk1 phosphorylation increased in the p*CTR4-2-FKS1* strain compared to that in H99. This increase was even more apparent upon treatment of the p*CTR4-2-FKS1* strain with caspofungin (Fig. 4A). In the presence of BCS, the amount of pMpk1 showed by the p*CTR4-2-FKS1* strain was similar to that of H99. The correlation between the level of *FKS1* expression and activation of CWIP is consistent with C. neoformans mounting a compensatory response to reduced Fks1 activity.

One of the components of the cell wall stress response mediated by the CWIP is increased deposition of chitin within the cell wall (36). To determine whether activation of the CWIP in the p*CTR4-2-FSK1* strain results in changes to the cell wall, we stained cells with calcofluor white (CFW) to characterize the amount of cell wall chitin. In general, we observed the typical chitin distribution on H99 cells grown in YPD, YPD plus 200 μM BCS, and YPD plus 50 μM copper sulfate, where staining is brightest at the septum, with less intense but uniform staining of the lateral cell wall (Fig. 4B and C). Interestingly, we observed a significant decrease in overall staining intensity upon treatment with 50 μM CuSO_4_. As expected, treatment of H99 with caspofungin resulted in a uniform increase in lateral cell wall staining (Fig. 4B/C).

Consistent with our Western blot data, we observed similar levels and distribution of chitin between the H99 and p*CTR4-2-FKS1* strains in the presence of BCS. Compared to BCS treatment, chitin levels increased slightly in YPD, with a uniform increase in lateral cell wall staining. However, with the addition of 50 μM copper sulfate, the chitin staining intensity of the p*CTR4-2-FKS1* strain increased significantly. Furthermore, the distribution of chitin was altered, with many cells displaying extremely bright staining of daughter cells or bright, nonuniform patches along the lateral cell wall. Treatment of the p*CTR4-2-FKS1* strain with caspofungin increased chitin amounts compared to those with YPD alone; however, the distribution in these cells more closely resembles that in YPD, with a uniform increase in chitin staining of the lateral cell wall. Together, these data are consistent with the conclusion that the copper-induced reduction in the expression of *FKS1* in the p*CTR4-2*-*FKS1* strain results in a functional reduction in Fks1 activity that subsequently activates the CWIP and a compensatory increase in chitin synthesis.

### Altered *FKS1* expression *in vivo* results in reduced fitness in a murine model of cryptococcosis.

**FIG 5:**
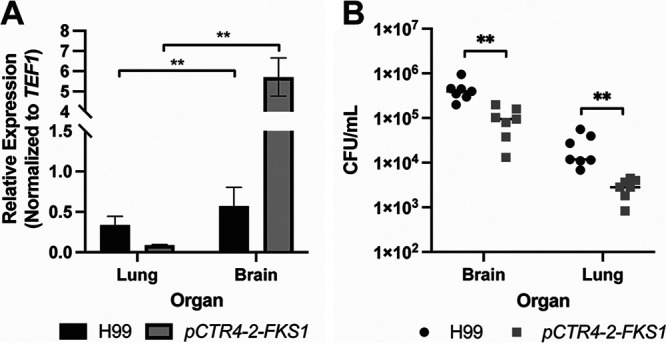
Repression or overexpression of *FKS1 in vivo* results in a fitness defect. (A) Expression of *FKS1* in the lungs and brains collected at 4 dpi from mice inoculated via lateral tail vein with the H99 or *pCTR4-2-FKS1* strains. Data represent mean and SEM of *FKS1* expression normalized to *TEF1*. *n* = 6 mice per group. **, *P* = 0.0022 by Mann-Whitney test, comparing the *pCTR4-2-FKS1* strain to the H99 strain for each organ. (B) Fungal burden of lungs and brains of mice collected at 4 dpi. Data represent individual values of 7 mice per group. **, *P* < 0.001 by Mann-Whitney test, comparing the H99 strain to the *pCTR4-2-FKS1* strain in each organ, corrected for multiple comparisons with a Bonferroni correction.

Next, we sought to determine whether the p*CTR4-2* expression system could be used to evaluate the effect of reduced *FKS1* expression on fitness during infection. To test this, we used the intravenous model of cryptococcosis, in which both the lungs and the brain become infected rapidly, resulting in reproducible fungal burden in both organs within 4 days. In this way, we could directly compare expression and fungal burden in the organs of the same animal. We inoculated AJ/cr mice with H99 or the p*CTR4-2-FKS1* strain via the lateral tail vein and then harvested brains and lungs at 4 days postinoculation. To confirm whether the expression of *FKS1* in the p*CTR4-2-FKS1* strain responds to the copper environment of each organ, we measured *FKS1* expression in both the lung and the brain. In accordance with *CTR4* expression data (Fig. 1), the expression of *FKS1* in the lungs was significantly reduced (*P* = 0.0022; by Mann-Whitney test) in the p*CTR4-2-FKS1* strain compared to that in H99 (Fig. 5A). The reduction in *FKS1* expression *in vivo* was not as pronounced as that observed *in vitro* (Fig. 2C) but was consistent with the expression of *CTR4 in vivo* (Fig. 1). Conversely, *FKS1* expression in the brain was significantly increased in the in p*CTR4-2-FKS1* strain relative to that in H99 (Fig. 5A; *P* = 0.0022 by Mann-Whitney test), which again follows the trend observed for *CTR4* expression (Fig. 5A). These data are consistent with the notion that the lungs are copper replete while the brain is copper limited and the copper-regulatable promoter can be used to modulate the expression of genes in murine models of infection.

Finally, we measured the fungal burden in lung and brain tissues of mice inoculated with H99 or the p*CTR4-2-FKS1* strain to determine if modulation of *FKS1* expression *in vivo* results in a fitness cost. In the lungs, where *FKS1* expression is significantly reduced in the p*CTR4-2-FKS1* strain compared to that in H99, we observed an ~1 log_10_ reduction in fungal burden (Fig. 5B). Despite the expression of *FKS1* in the p*CTR4-2-FKS1* strain being much closer to that in H99 *in vivo* compared to *in vitro*, the fitness defect is much more pronounced *in vivo*. This suggests that maintenance of *FKS1* activity is much more important for C. neoformans survival *in vivo* than that *in vitro*.

While overexpression of *FKS1 in vitro* had no effect on fitness, the fungal burden of brains inoculated with the p*CTR4-2-FKS1* strain was reduced by about 0.5 log_10_ compared to that in those inoculated with H99 (*P* = 0.0012 by Mann-Whitney test), suggesting that overexpression of *FKS1 in vivo* is detrimental to C. neoformans growth or survival in the brain (Fig. 5B). Interestingly, we observed the same results with control experiments performed with the p*CTR4-2-FAS1* strain studied by the Perfect lab; specifically, overexpression of *FAS1* reduced fungal burden in the brain (see Fig. S1 in the supplemental material). The Perfect lab also reported that the brain burden for this strain was reduced after dissemination from the lung. Together, these data support the hypotheses that the regulation of *FKS1* expression is required for full fitness *in vivo* and that reduced *FKS1* activity is more important for fitness during infection than under standard *in vivo* conditions.

10.1128/msphere.00163-22.1 FIG S1 The fungal burden of p*CTR4-2-FAS1* is significantly lower in both the brain and the lung during infection. Fungal burden of brains and lungs of mice collected 4 days postinoculation via the lateral tail vein. Data represent 7 mice per group; control (H99) data are the same data shown in Fig. 4A. **, *P* = 0.0006 by Mann-Whitney test corrected for multiple comparisons with a Bonferroni correction. Download FIG S1, PDF file, 0.1 MB. Copyright © 2022 Beattie et al. 2022 Beattie et al. https://creativecommons.org/licenses/by/4.0/ This content is distributed under the terms of the Creative Commons Attribution 4.0 International license.

## DISCUSSION

The goals of this study were 2-fold, as follows: (i) to explore the scope of copper-regulated gene expression as an approach to studying essential genes in C. neoformans infection models and (ii) to characterize the effect of reduced expression of the putatively essential gene *FKS1 in vitro* and *in vivo*.

To our knowledge, this is the fourth gene that has been studied *in vivo* using the *CTR4-2* promoter to modulate expression (15, 18). Although this approach has utility, there are some important limitations to the system that must be considered during experimental design and data interpretation. First, this system should always be evaluated on a gene-to-gene basis. For any given gene, the wild-type levels of expression and the amount of repression needed to see a phenotype can vary. For example, a gene with very low expression may already be expressed at levels similar to *CTR4* expression under copper-replete conditions and may not be repressed *in vivo* to the point where robust phenotypes can be detected. Conversely, highly expressed genes may still be “repressed” under copper-depleted conditions; in such cases, the p*CTR4-2*-regulated allele will represent a knockdown in both conditions. Second, it is critical that the expression of the target gene be checked *in vitro* and *in vivo* to ensure that gene expression is responding to copper concentration. When manipulating essential genes, chromosomal rearrangements and/or mutations can occur during strain generation or as the strain is passaged in routine lab use. These alterations could result in unexpected expression patterns. Third, because the copper concentrations cannot be changed in the animal, this promoter system is limited to the study of the effect of reduced gene expression during the establishment and progression of pulmonary infection or, in some cases, to assess the effects of gene overexpression in the brain. With these limitations in mind, however, this system can be a powerful tool for the evaluation of essential C. neoformans genes *in vivo.*

We chose to further explore the *in vivo* utility of the *pCTR4-2* promoter system by applying it to the β-1,3-glucan synthase gene *FKS1* to learn more about the effect of reduced expression on C. neoformans *in vitro* and *in vivo*. As briefly outlined in the introduction, C. neoformans is notably resistant to echinocandins, with MIC values orders of magnitude higher than those for other pathogenic yeasts. Previous genetic experiments indicate that *FKS1* is essential and that the enzyme, Fks1, is inhibited by echinocandins in a manner similar to that for enzymes isolated from susceptible species (22, 37). Gold particle immune-electron microscopy studies with antibodies specific to 1,3-β-glucan linkages indicate that C. neoformans cells contain this polymer and that treatment with an echinocandin reduces their levels in the cells (36). Consistent with other yeasts, exposure of C. neoformans to echinocandins also activates the cell wall stress response mediated by the CWIP (Fig. 4A) (35). The strong correlation between the effects of echinocandins on susceptible yeast species and on C. neoformans suggests that the mechanism of action of the drug is similar among the discordant organisms.

Our studies with the p*CTR4-2-FKS1* strain make further contributions to our understanding of the phenotypes associated with reduced glucan synthase expression. First, suppression of *FKS1* expression leads to activation of the CWIP as determined by increased phosphorylation of Mpk1 under those conditions. This activation is seen in other pathogenic fungi as well (38). Second, suppression of *FKS1* expression increases the susceptibility of the strain to caspofungin, although it was reduced by only 4-fold despite a 100-fold reduction in *FKS1* expression. In C. albicans, overexpression of *FKS1* did not increase the MIC of caspofungin toward planktonic cells; it did, however, increase the MIC in C. albicans biofilms (39). Third, cells treated with either caspofungin or that have copper-reduced expression of *FKS1* show increased chitin within the cell wall (Fig. 4C and D). Chitin has been shown to be a key modulator of echinocandin tolerance and resistance (40). Fourth, the addition of an osmotic stabilizer (1 M sorbitol) to the medium reduces the effect of reduced *FKS1* expression on growth. This phenomenon is well described for a range of cell wall-targeting agents and fungal pathogens (36). Thus, the phenotypes associated with the genetic reduction in *FKS1* expression correlate quite well with the effects of caspofungin exposure in other yeast.

A key finding of our work is that the C. neoformans tolerates a dramatic reduction in *FKS1* expression without significant changes in growth/viability *in vitro*. This raises the question of what mediates this tolerance. First, it is possible that only a very small amount of transcript is required to maintain 1,3-β-glucan synthase protein at levels needed to preserve viability. Kalem et al. has shown that the regulation of *FKS1* mRNA stability and translation plays an important role in resistance to caspofungin (31). Second, it is possible that the Fks1 protein is remarkably stable and that there is little correlation between gene and protein expression. If the difference in expression were only a fewfold then this would seem to be the much more likely explanation; since the difference is 2 orders of magnitude, it is very difficult to invoke this mechanism. Again, Kalem et al. found that that there was only an ~1.5-fold difference between mRNA and Fks1 protein levels in a mutant with increased translation of *FKS1* message (31).

A third explanation for our observations is that the cell wall stress-induced compensatory response triggered by reduced Fks1 activity in C. neoformans is much more effective than that for other organisms. A substantial number of genes not directly related to glucan synthesis have been shown to play important roles in the resistance of C. neoformans to echinocandins (28–31). These may combine with the CWIP-mediated responses to effectively buffer the cell against reduced Fks1 activity/*FKS1* expression in a manner not observed for other yeasts. This would explain the apparent disconnect between the essential nature of the *FKS1* gene (21) and the tolerance of C. neoformans to all but a presumably nearly complete depletion/inhibition of 1,3-β-glucan synthase activity. Kraus et al. have suggested that the calcineurin and the CWIP effectively compensate for reduced 1,3-β-glucan synthase activity in the absence of other stressors (35). As part of their studies, Kraus et al. found that *FKS1* expression is induced by activation of the CWIP. Our data suggest that increased expression of *FKS1* is unlikely to have a significant role in the response to reduced 1,3-β-glucan synthase activity and that other mechanisms, such as altered chitin homeostasis, appear to be more important. The altered chitin content of strains with reduced *FKS1* expression and the role of chitin in the response to lowered 1,3-β-glucan synthase activity further support that hypothesis.

Interestingly, we observed that treatment of H99 with 50 μM copper sulfate results in a significant reduction in overall chitin levels. Links between the cell wall and copper homeostasis have been postulated previously, as the copper-responsive transcription factor Cuf1 regulates cell wall synthesis genes (41). Recently, Probst et al. solidified a connection between copper homeostasis and the cell wall, showing that mutation of the copper homeostasis machinery results in changes to the levels of chitin in the cell wall (42). Similarly to our results reported here, Probst et al. show calcofluor white images under copper sufficiency conditions with less intense staining than those in observed in copper-deficient medium.

We also tested the ability of this strain to establish infection in a murine model of cryptococcosis. In both the brain and lungs, the modulation of expression of *FKS1 in vivo* by copper resulted in a significant reduction in fungal burden. As we expected, in the copper-replete lungs, the expression of p*CTR4-2-FKS1* was significantly reduced, resulting in an ~1 log_10_ reduction in fungal burden compared to that in animals inoculated with H99. In the copper-deplete brain, the expression of *FKS1* was significantly increased in the p*CTR4-2-FKS1* strain compared to that in H99; however, we observed a half-log reduction in fungal burden. These results support the idea that proper regulation of *FKS1* and the cell wall composition is critical for full fitness *in vivo*. Although we did not see any associated defects *in vitro*, the environment *in vivo* is much more complex and dynamic. For example, copper is subject to regulation by the host as part of nutritional immunity (43), and thus copper concentrations within the microenvironment of fungal lesions can change rapidly. In Mycobacterium tuberculosis, copper has been shown to accumulate at granulomatous lesions in the lung (44). In addition, macrophages use copper as an antimicrobial by pumping high levels of copper into phagolysosomes, resulting in higher copper exposure to cells within macrophages. In addition, even slight changes to the fungal cell wall can have profound impacts on disease pathology since the cell wall is the interface between fungal and host cells and perturbations in cell wall architecture often leave pathogen recognition patterns exposed.

Finally, we note that reduction in *FKS1* expression by ~4-fold results in a 1 log_10_ reduction in fungal burden, while *in vitro* the growth is only minimally affected. This suggests that the compensatory mechanisms that support the ability of C. neoformans to tolerate reduced 1,3-β-glucan synthase expression are much less effective *in vivo*. One explanation of this finding is that C. neoformans cells are subject to a combination of stressors that all must be managed through the same compensatory mechanisms. Although further work will be required to understand the contributing factors and mechanisms, it is clear that 1,3-β-glucan synthase expression is much more important *in vivo* than would be predicted from *in vitro* phenotypes.

This work demonstrates the utility of the copper-regulated promoter p*CTR4* in assessing the role of essential genes in animal models of infection, a critical step in target validation for the development of novel antifungal drugs. Furthermore, we have begun to further unravel the mystery of caspofungin resistance in C. neoformans. Our data support the idea that C. neoformans can tolerate a significant reduction in Fks1 activity, resulting in reduced efficacy of caspofungin despite direct inhibition of the enzyme itself. A complete understanding of how the cell wall architecture and response to cell wall stress differs between C. neoformans and other pathogenic fungi can inform the development of cell wall-active antifungals and understanding of how C. neoformans interfaces with the host during disease.

## MATERIALS AND METHODS

### Strains, media, and chemicals.

All experiments were performed with C. neoformans H99 Stud. C. neoformans strains were maintained on YPD and stored at −80°C in 25% glycerol. Plasmid pCTR4-2 was generously provided by Tamara Doering (Washington University, St. Louis, MO). Plasmids BHM2329 and BHM2403 were generously provided by Hiten Madhani (University of San Francisco, San Francisco, CA). Copper sulfate pentahydrate (catalog no. C7631), bathocuproinedisulfonic (BCS; catalog no. 146625) acid disodium salt hydrate, and caspofungin diacetate (catalog no. SML0425) were obtained from Sigma.

### Construction of plasmids.

To make pUL-HYG, pZY97 (45) was simultaneously digested with SacI and AatII (catalog no. R0157 and R0117; New England Biolabs) to drop out the existing resistance cassette and generate the cut vector for Gibson assembly. The hygromycin resistance (HYG^r^) cassette was amplified from pXL1-pTEF1 (46) with primers LCR033 and LCR012, designed to overlap the cut vector by 20 bp to facilitate Gibson assembly and add a 20-bp universal linker at each end of the cassette. Cut vector (100 ng) with a 2-fold molar excess of insert were combined in the Gibson assembly reaction (catalog no. E5510S; New England Biolabs), following the manufacturer’s instructions. Plasmids were isolated from individual clones, and the universal linker region was confirmed at each end of the resistance cassette by Sanger sequencing with primers LCR033 and LCR034. Functionality of the inserted resistance cassette was confirmed by transformation into H99 and selection on 400 mg/mL hygromycin plates.

We generated plasmid pSB-CTR4 containing the pCTR4-2 promoter downstream of HYG^r^ to facilitate promoter replacement of additional genes by simply amplifying the resistance cassette and pCTR4-2 promoter with primers containing short-arm homology to a gene of interest. The promoter replacement construct was generated by amplifying the pCTR4-2 promoter from plasmid pCTR4-2 (17) with primers SP181 and SP196 to add homology to the HYG^r^ cassette at the 5′ end of the product and homology to pUC19 at the 3′ end of the product. The HYG^r^ cassette was amplified with primers SP197 and SP180 from pUL-HYG. The 5′ end of this product contains homology to pUC19. These fragments were cloned with InFusion cloning (catalog no. 638911; TaKaRa) into pUC19 with the HYG resistance cassette upstream of pCTR4-2. Plasmids were isolated from individual clones and confirmed by digestion with HindIII (catalog no. R0104; New England Biolabs), and the resulting plasmid pSB-pCTR4 was used to amplify the final *FKS1* promoter replacement cassette.

### Construction of pCTR4-2-FKS1.

pCTR4-2-FKS1 was generated using CRISPR/Cas9 with short homology repair (32). CnoCas9 was amplified using SP29 and SP30 from BHM2403 (32). The single guide RNA (sgRNA) was designed using the Eukaryotic Pathogen CRISPR guide RNA Design tool (47). To generate the complete sgRNA construct, (i) SP106 and SP200 and (ii) SP105 and SP201 were used to amplify the U6 promoter and sgRNA scaffold, respectively, with the 20-nucleotide (nt) guide sequence from plasmid BHM2329. These two fragments were joined with overlap extension PCR with primers SP31 and SP32.

The promoter replacement construct was amplified from pSB-pCTR4 with primers SP199 and SP202, which contained 50 bp of microhomology to the *FKS1* 5′ untranslated region (UTR). Electroporation-mediated transformation was used to transform 1 μg Cas9, 1 μg guide RNA (gRNA), and 3 μg of p*CTR4-2-FKS1* promoter construct (48), and cells were plated on YPD with 400 μg/mL hygromycin B (catalog no. J69681; Alfa Aesar). Transformants were verified with PCR spanning the joint between the promoter construct and the *FKS1* coding sequence and were tested for response to copper by growth assay and gene expression.

### Antifungal susceptibility and interaction assays.

MICs were determined using modified CLSI guidelines (49). Yeasts were cultured overnight in 3 mL YPD at 30°C, then washed twice in sterile phosphate-buffered saline (PBS). Twofold serial dilutions of each drug were prepared in RMPI plus morpholinepropanesulfonic acid (MOPS; pH 7) (Gibco RPMI 1640 with l-glutamine [catalog no. 11875-093] and 0.165 M MOPS), and then 1 × 10^3^ cells were added per well in a total volume of 200 μL. Plates were incubated at 37°C for 72 h.

For fractional inhibitory concentration assays, standard checkerboard assays were performed as previously described (50). Briefly, 2-fold dilutions of caspofungin or CuSO_4_ were prepared in YPD at 4-fold the desired final concentration. Aliquots (50 μL) of the caspofungin dilution series were dispensed into a 96-well plate with the concentration decreasing across the columns. Next, 50 μL of the CuSO_4_ dilution series was dispensed into the plate with the concentration decreasing down the rows, such that the top corner of the plate contained the highest concentration of each compound and the opposite corner contained vehicle only. An aliquot of 1 × 10^3^ cells was added per well for a total volume of 200 μL. Plates were incubated for 42 h at 37°C, and then the MICs of each drug alone those of each drug in combination were determined.

### Plate-based growth assay.

Overnight cultures of H99 and the *pCTR4-2-FKS1* strain were diluted to an optical density at 600 nm (OD_600_) of 1, and then 10-fold serial dilutions were prepared in PBS. A 5-μL aliquot of each dilution was spotted on the indicated medium, then incubated for 48 to 72 h at 30°C or 37°C. Plates were imaged using an Epson Perfection V600 Photo Scanner. Contrast was adjusted equally across all images for the best visualization of colonies.

### Characterization of Mpk1 phosphorylation by Western blot analysis.

Overnight cultures of H99 and the *pCTR4-FKS1* strain were diluted to an OD of 0.1 in YPD, YPD plus 50μM CuSO4, or YPD plus 200μM BCS, and then grown to the mid-log phase (4 h) at 30°C with shaking at 200 rpm. Protein was extracted in extraction buffer (10 mM HEPES [pH 7.4 to 7.9], 1.5 mM MgCl_2_, 10 mM KCl, 1 mM dithiothreitol [DTT], 1× HALT protease, and phosphatase inhibitor cocktail [catalog no. 1861280; Thermo Scientific]) by five bead-beating cycles of 30 s followed by 30 s on ice per cycle. Debris and beads were pelleted before supernatant was recovered, and the protein concentration was quantified by Bradford assay. Protein (20 μg) was loaded on a 10% SDS-PAGE gel and run at 80 V. Samples were transferred to nitrocellulose membrane for 1 h at 100 V, and then the membrane was stained with Ponceau for 5 min at room temperature (RT). The membrane was blocked with 5% bovine serum albumin (BSA) in Tris-buffered saline with Tween 20 (TBST) for 1 h at RT, and then the membrane was incubated with 1:2,000 rabbit anti-p-p44 (phospho-p44/42 MAPK, catalog no. 4370; Cell Signaling) in 5% BSA/TBST overnight at 4°C. The membrane was washed 3 times for 5 min with TBST, then incubated for 1 h at RT with 1:10,000 goat anti-rabbit horseradish peroxidase (HRP) (catalog no. STAR208P; Bio-Rad). The membrane was washed 3 times for 5 min with TBST, then developed with chemiluminescent substrate and imaged on a Thermo Scientific myECL imager.

### Microscopy-based characterization of chitin content and distribution.

Cells were cultured under the same conditions described above. For calcofluor white (CFW), cells were washed twice with PBS, then resuspended in 10 μg/mL CFW in PBS (Fluorescent Brightener 28, catalog no. F3543; Sigma) and incubated for 20 min at RT in the dark. Cells were imaged on a Nikon epifluorescence microscope with a CoolSnap HQ2 camera and Nikon Elements image acquisition and analysis software. The mean pixel intensity of images was measured using ImageJ. Images included in the figure were processed in Photoshop only to increase ease of viewing. All images were adjusted equally.

### *In vitro* quantitative RT-PCR.

Overnight cultures of the H99 and pCTR4-FKS1 strains were diluted to an OD of 0.1 in YPD, YPD plus 50μM CuSO4, or YPD plus 200μM BCS and then grown to the mid-log phase (4 h) at 30°C with shaking at 200 rpm. Cells were collected, and pellets were frozen at −80°C and then lyophilized. Dried tissue was homogenized with zircon silica beads using the Fast-Prep24 homogenizer catalog no. 116004500; MP Biomedicals). RNA was isolated using the PureLink RNA kit (catalog no. 12183025; Invitrogen) according to the manufacturer’s protocol, and then 5 μg of RNA was treated with the Turbo DNA-free kit (catalog no. AM1907; Invitrogen). A 500-ng sample of RNA was used for cDNA synthesis with the iScript cDNA synthesis kit (catalog no. 1708840; Bio-Rad), and cDNA was diluted 1:5 for quantitative RT-PCR (qRT-PCR). A 2-μL aliquot of diluted cDNA was used per reaction with iQ SYBR green Supermix and 0.20 μM primers. qRT-PCR was performed on the Bio-Rad CFX Connect using a 3-step amplification with 54°C annealing temperature and melt curve analysis. Primers are listed in Table S1 in the supplemental material.

10.1128/msphere.00163-22.2 TABLE S1 Primers used in this study. Download Table S1, PDF file, 0.1 MB. Copyright © 2022 Beattie et al. 2022 Beattie et al. https://creativecommons.org/licenses/by/4.0/ This content is distributed under the terms of the Creative Commons Attribution 4.0 International license.

### *In vivo* quantitative PCR.

Female A/J mice (6 weeks old; Jackson Laboratory) were inoculated with 5 × 10^4^ CFU/mL in 200 μL PBS via the lateral tail vein or with 1 × 10^6^ CFU/mL in 50 μL PBS via intranasal instillation. Brains and lungs were collected at 4 and 8 days postinoculation and then lyophilized. Tissue was homogenized with 2.3-mm zirconia-silicate beads using the Fast-Prep24 homogenizer (catalog no. 116004500; MP Biomedicals). 1 mL of TRIzole (catalog no. 15596026; Invitrogen) was added to each sample and incubated at RT for 10 min. Samples were clarified by spinning at 10,000 × *g* for 5 min, then supernatant was transferred to a new tube and 200 μL of chloroform was added with mixing by inversion. Samples were spun at 12,000 × *g* for 15 min, then the aqueous phase was transferred to a gDNA removal column (RNeasy Plus minikit, catalog no. 74136; Qiagen) then RNA was purified following the manufacturer’s instructions. A 500-ng sample of RNA was used for cDNA synthesis with the iScript cDNA synthesis kit (catalog no. 1708840; Bio-Rad), and cDNA was diluted 1:1 for qRT-PCR. A 2-μL aliquot of diluted cDNA was used per reaction with iQ SYBR green Supermix and 0.20 μM primers. qRT-PCR was performed on the Bio-Rad CFX Connect using a 3-step amplification with 54°C annealing temperature. We observed no amplification with our primer sets on cDNA prepared from mice inoculated with PBS. Primers used are listed in Table S1.

### Determination of fungal burden in a mouse model of disseminated cryptococcosis.

Female A/J mice (6 weeks old; Jackson Laboratory) were inoculated with 5 × 10^4^ CFU/mL in 200 μL PBS via the lateral tail vein. Brains and lungs were collected at 4 days postinoculation and homogenized in PBS with a benchtop homogenizer (VWR). Homogenates were diluted in a 10-fold dilution series, and each dilution was plated on YPD. Plates were incubated at 30°C for 48 h, and colonies were counted. Each group contained 7 mice per group. Differences in fungal burden between groups were analyzed using the Mann-Whitney test in GraphPad Prism 9.

### Ethics statement.

The Guide for the Care and Use of Laboratory Animals of the National Research Council was strictly followed for all animal experiments. The animal experiment protocols were approved by the Institutional Animal Care and Use Committee at the University of Iowa (protocol no. 7102064).
