# Supplementary material for: FKS1 Is Required for Cryptococcus neoformans Fitness In Vivo: Application of Copper-Regulated Gene Expression to Mouse Models of Cryptococcosis
Source: mSphere. 2022 May 4;7(3):e00163-22. doi: 10.1128/msphere.00163-22 (PMC9241531; doi:10.1128/msphere.00163-22)
Supplement: FIG S1 [file msphere.00163-22-s0001.pdf]

Supplementary Material

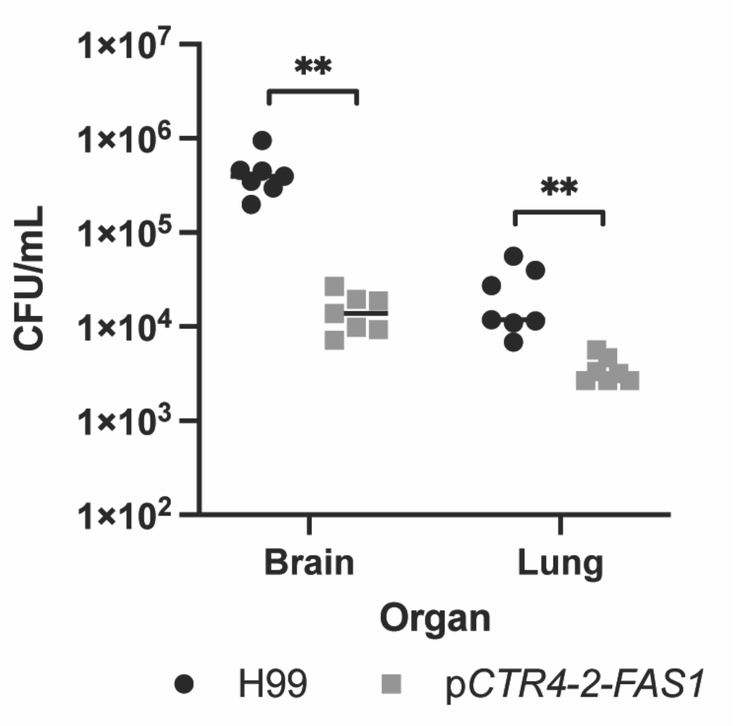

Figure S1. **The fungal burden of p*CTR4-2-FAS1* is significantly lower in both the brain and the lung during infection**. Fungal burden of brains and lungs of mice collected 4 days post-inoculation via the lateral tail vein. Data represent 7 mice per group; control (H99) data is the same data shown in Figure 4A. **p=0.0006 by Mann-Whitney test corrected for multiple comparisons with a Bonferroni correction.
