# Supplementary material for: FKS1 Is Required for Cryptococcus neoformans Fitness In Vivo: Application of Copper-Regulated Gene Expression to Mouse Models of Cryptococcosis
Source: mSphere. 2022 May 4;7(3):e00163-22. doi: 10.1128/msphere.00163-22 (PMC9241531; doi:10.1128/msphere.00163-22)
Supplement: TABLE S1 [file msphere.00163-22-s0002.pdf]

Primer Name	Description	Sequence
SP200	Fks1 gRNA5 Fwd	GACCTGTGAATACGCTACCGGTTTTAGAGCTAGAAATAGCAAG
SP201	Fks1 gRNA5 Rev	CGGTAGCGTATTCACAGGTCACAGTATACCCTGCCGGTG
SP106	gRNA 3' flank Rev	TCATTAGGCACCCCAGGC
SP105	gRNA 5' Flank Fwd	TCATCGCTATTACGCCAGC
SP31	U6 promoter Fwd	CCATCGATTTGCATTAGAACTAAAAACAAAGCA
SP32	gRNA scaffold Rev	CCGCTCGAGTAAAACAAAAAAGCACCGAC
SP29	Cas9 For	GGTGACGCTGTGAGAGTGG
SP31	Cas9 Rev	GGGCCCCTCTTCACGTGG
SP201	Fks1 microhomology Fwd	GAAAAGGAAGCAGACCCTTTCGGCGGTGGCGGGGGATTGGGATATGACATAGCTCGGTACCCTGGATTG
SP199	Fks1 microhomology Rev	ATTCCTTGAACGAAAACGTAAAACAGTAACTGCCTCGTGATTTGAGGGTACGGTCGCCTCAAACAATGC
SP188	CTR4 RT Fwd	CCT CGG GAT GTA CTT CAA
SP189	CTR4 RT Rev	TCC TCC ACC ACA AGT ATC
SP75	TEF1 RT Fwd	CGT CAC CAC TGA AGT CAA GT
SP76	TEF1 RT Rev	AGA AGC AGC CTC CAT AGG
SP203	FKS1 RT Fwd	CCC ATG TTT ATC CCT TAC
SP204	FKS1 RT Rev	CCT GAA GCT GTA AAT AGG
LCR011	UL-HYG Fwd	TCAATCCAATCAGTGACGTCACGGTCGCCTCAAACAATGCTTGCTGCGAGGATGTGAGC
LCR012	UL-HYG Rev	GCCAAGCTTGGTACCGAGCTCCTGAGAGGAGGCACTGATGCAGTGTGCTGGAATTCGCCC
LCR033	UL Sequencing 5'	ATCCGGATATGCAGAAACTCATCC
LCR034	UL Sequencing 3'	AACAGCTATGACCATGATTACGCC

Table S1. Primers used in this study
